# Congenital midline cervical cleft: a case report

**DOI:** 10.1186/s13256-019-2116-6

**Published:** 2019-06-09

**Authors:** Mazhar Çelikoyar, Erkan Aktan, Gülen Doğusoy

**Affiliations:** 1 Department of Otolaryngology, Istanbul Florence Nightingale Hospital, Abide-i Hürriyet Cad. No:166 34381, Sisli, Istanbul, Turkey; 2 Department of Pathology, Gayrettepe Florence Nightingale Hospital, Cemil Aslan Güder Sk, No: 834349, Besiktas, Istanbul, Turkey

**Keywords:** Midline cleft, Congenital midline cervical cleft, Surgical excision, Z-plasty, Congenital neck lesions

## Abstract

**Background:**

Midline cervical cleft is a very rare congenital anomaly. According to a literature search, until 2014 only 205 cases were reported.

**Case presentation:**

We present a classic case of congenital midline cervical cleft. This was a case of a 3-year-old Middle Eastern boy. The lesion was excised and the defect was closed via multiple Z-plasties.

**Conclusions:**

Midline cervical cleft, although a rarity, when presented needs surgical treatment, which comprises surgical excision and closure that lessens the possibility of scar visibility and contracture.

## Introduction

Midline cervical cleft (MCC) is a rare congenital anomaly. According to a literature search, by 2014 only 205 cases had been reported [1]. The first recorded case of MCC was in 1848 by Luschka, while Bailey documented the first description of this abnormality in 1924 [2, 3]. It is an entirely sporadic lesion that is only occasionally associated with other developmental defects including bifid mandible and microgenia, clefting of the sternum, and a possible loss of other midline structures, such as portions of the hyoid bone [4, 5].

It is not considered a true cleft because no skin gap exists. It represents a variant of the cleft category number 30 of the Tessier classification system of craniofacial defects [2, 6, 7].

The clefts have a remarkably consistent appearance and are typically noticed at birth as a defect at the ventral aspect of the neck. Superiorly, there is a skin tag with the inferior margin being formed by a short sinus, normally approximately 1 cm in length. A mucosal surface divides the two. No antenatal problems are generally identified and Caucasian women seem to be the most frequently affected [8].

MCC is diagnosed by clinical examination, and the main characteristic is a protruding lesion at the midline of the anterior region of the neck, between the chin and the suprasternal notch [3]. The cardinal diagnostic features are: (a) fistula opening is located caudally, (b) intermittent serous fluid discharge in the early neonatal period, (c) nipple-like appearance of the cleft in the superior aspect, and (d) widened scar and minimal neck contracture in later life [6].

Treatment is surgical excision, which is required largely to improve the cosmetic appearance of the neck and to avoid contracture formation of the ventral neck [8, 9]. We present a classic case of MCC treated with excision and defect closure via multiple Z-plasties.

## Case presentation

**Fig. 1:**
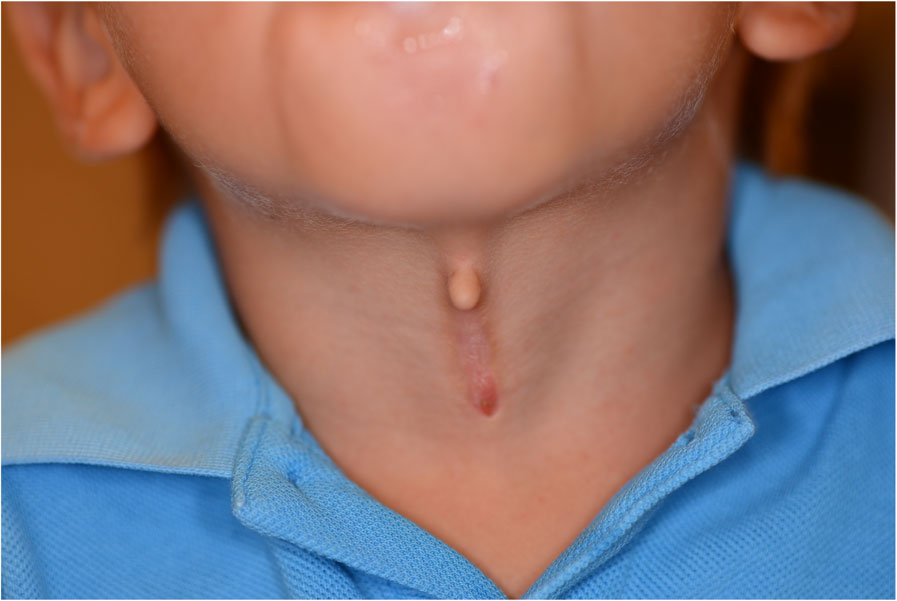
Preoperative appearance of the patient’s neck

A 3-year-old Middle Eastern boy presented with a defect in the midline of his neck. He was born at full term by normal vaginal delivery and had no significance in his past medical history. There was no family history of congenital defects or consanguinity. The anomaly was located in the ventral midline of his neck (Fig. 1). The superior aspect was composed of a skin tag leading to a short mucosa-like raw surface. Inferiorly, there was a sinus present with a greenish, thick residue occluding the opening. There was no contracture of the neck. He did not appear to be troubled by the lesion and a full examination was otherwise normal, except for adenoidal hypertrophy.

**Fig. 2:**
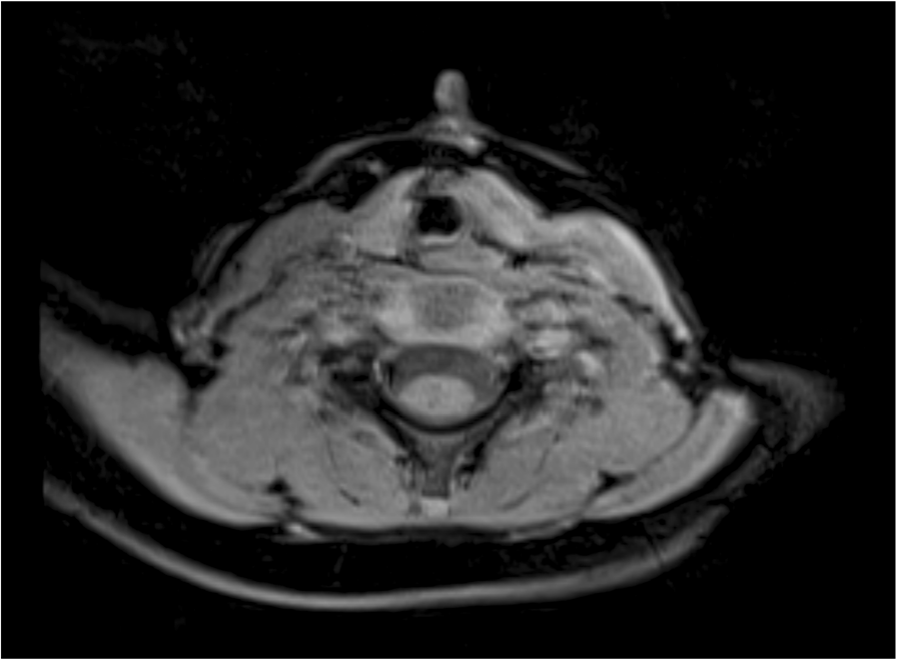
Magnetic resonance imaging view of the patient’s neck, from approximately 1 year prior to the surgery. Note the nipple that belongs to the lesion

He had an MRI done elsewhere, indicating a soft tissue mass without any fistula tract. Despite contrast material being injected through the opening at the caudal end of the lesion, the diagnosis of MCC was established. No evidence of any other neck anomaly was found (Fig. 2). The sinus, less than 1 cm in length, was found to extend caudally to the suprasternal notch. There were no attachments to underlying structures.

**Fig. 3:**
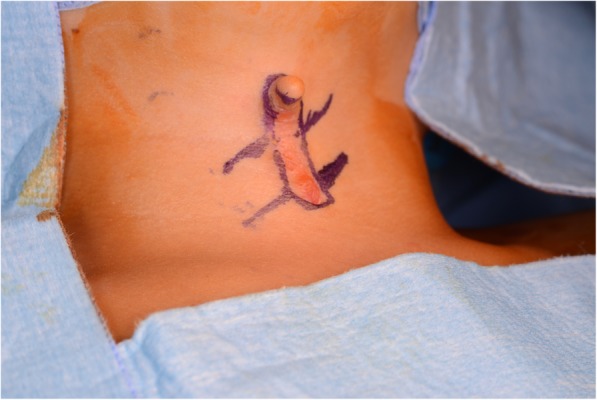
Surgical markings

**Fig. 4:**
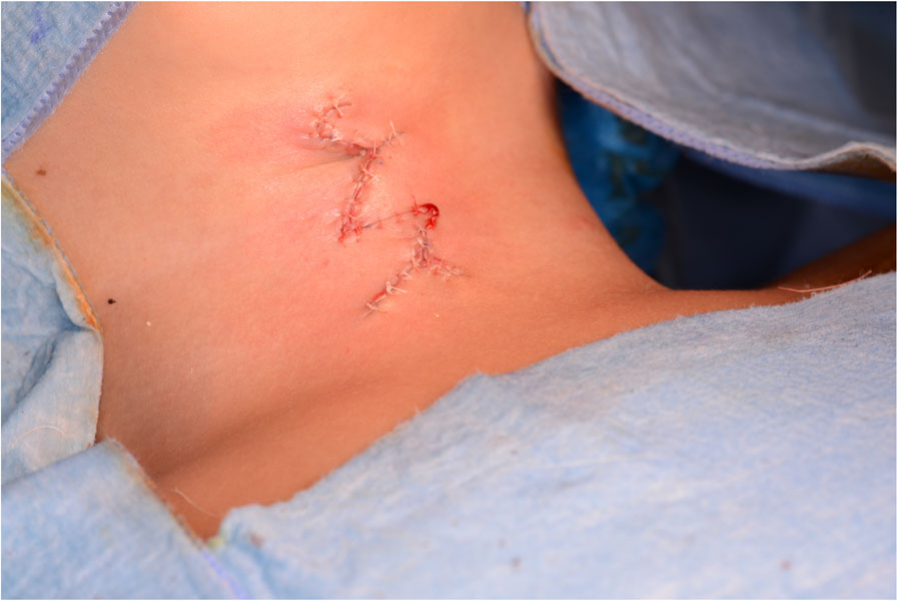
Closure of the wound has been accomplished

A surgical removal and immediate closure with multiple Z-plasties were performed. Surgical removal was done with an incision 1–2 mm from the periphery of the lesion, deepened down to the supraplatysmal plane (Fig. 3). During the surgery, the sinus at the caudal end of the lesion was probed and followed caudally until it ended, which was found to be approximately 2 cm long. This underdeveloped fistula tract ended right above the thymus gland. The cranial end of the defect had a fibrous band extending up to the mandible and this band was resected together with the cervical lesion. The midline lesion was found to be superficial and hence the excision was done at the subdermal level. A double Z-plasty was found to be sufficient for the closure. Closure was done with 5–0 vicryl interrupted sutures at the subcutaneous level and 6–0 rapid vicryl interrupted sutures for skin closure (Fig. 4).

**Fig. 5:**
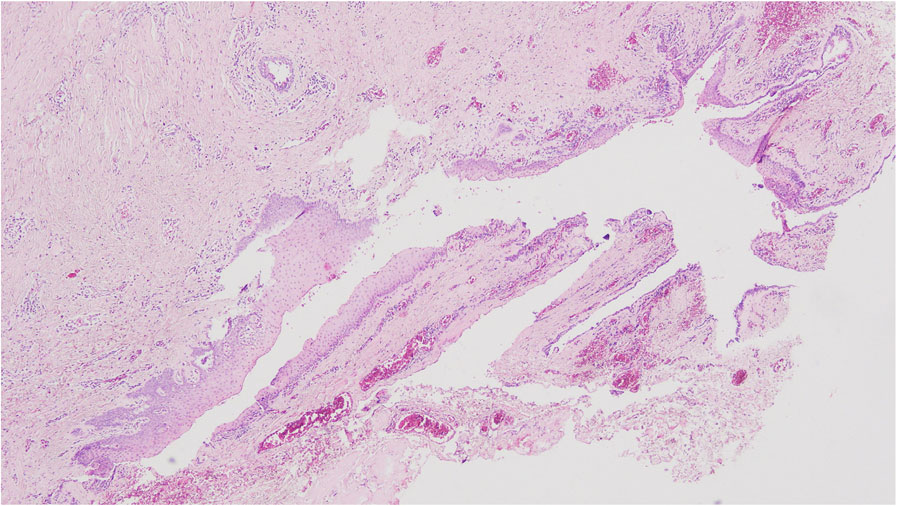
Histologic appearance of the cleft. Stratified squamous epithelial cells are seen covering the cleft with few adnexial structures at the dermal level. Hematoxylin and eosin staining was used

A pathological examination of the specimen confirmed our clinical diagnosis. The findings were consistent with stratified squamous epithelial cells covering the cleft with few adnexial structures at the dermal level (Fig. 5).

**Fig. 6:**
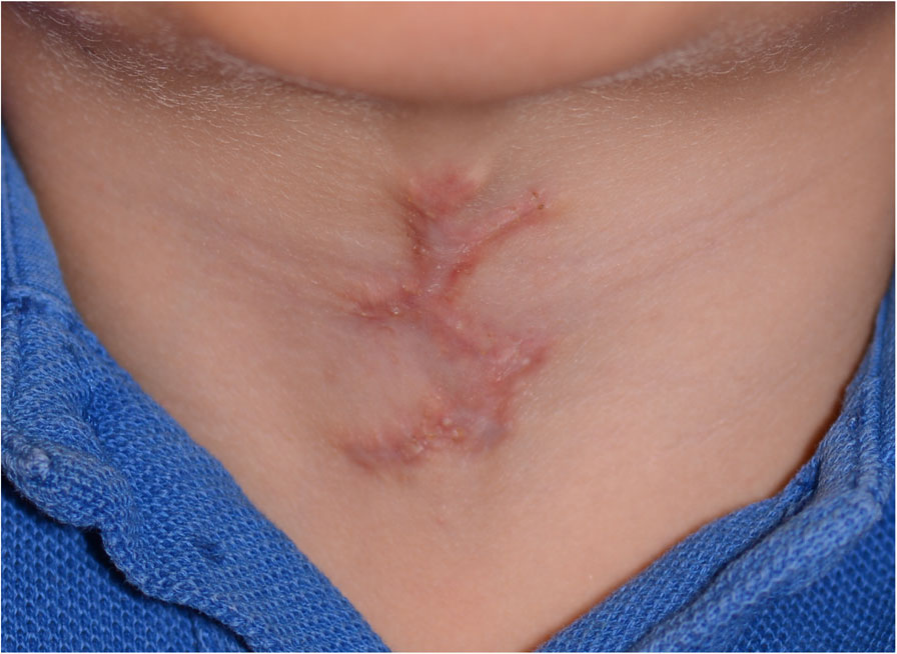
One-month postoperative appearance of the patient’s neck

One month follow-up examination revealed an uneventful healing period, with redness along the incision scar and some nodularities, which were most probably due to the subcutaneous suture material. He was able to move his head in all directions without any restriction or pain (Fig. 6).

**Fig. 7:**
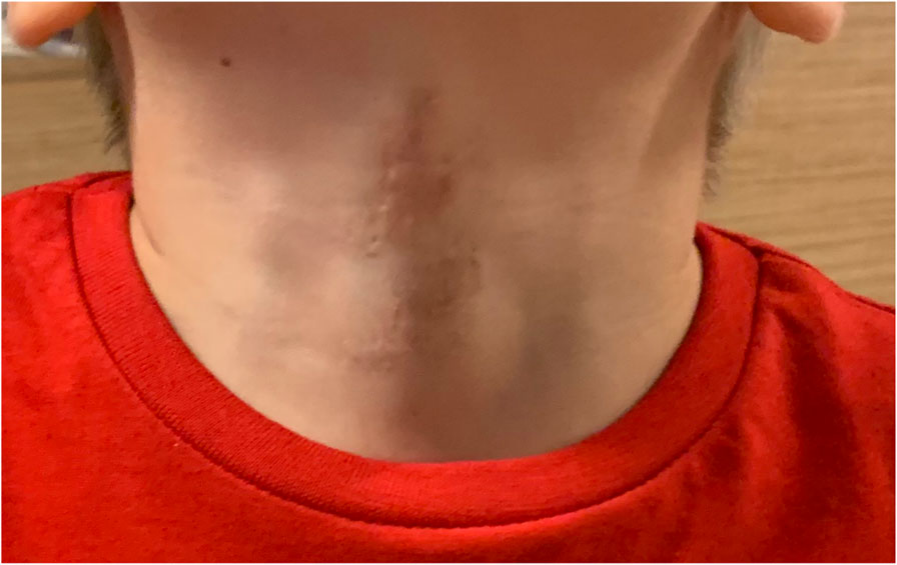
Fourteen-month postoperative appearance of the patient’s neck

**Fig. 8:**
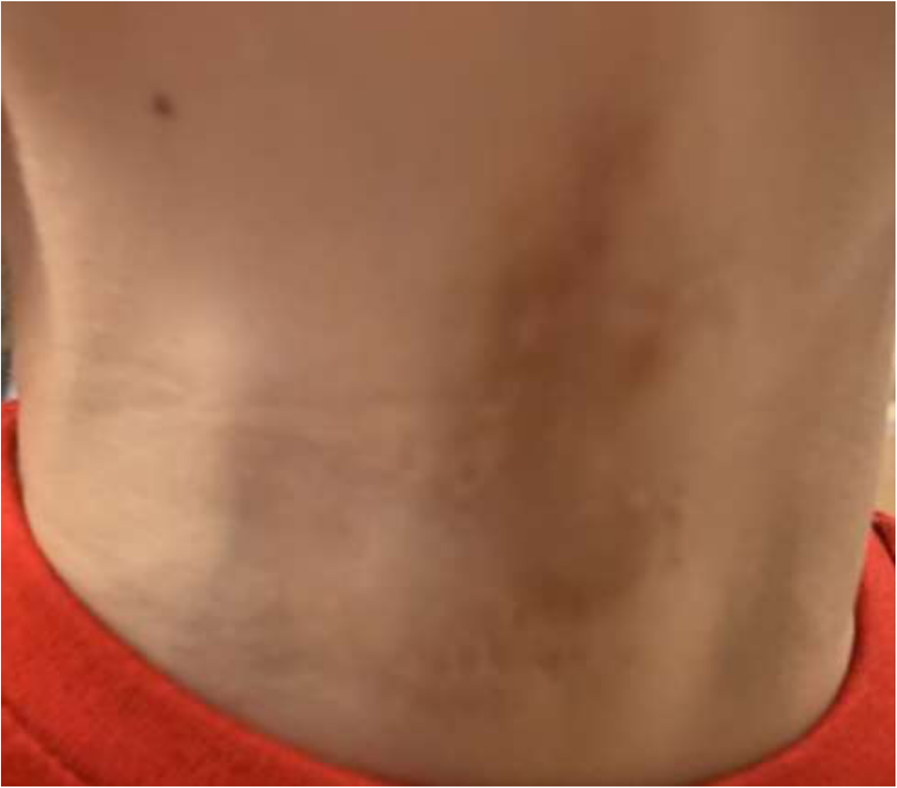
Fourteen-month postoperative appearance of the patient’s neck (close up)

A 14-month follow-up examination showed an acceptable level of scarring causing no restriction of neck movements (Figs. 7 and 8).

## Discussion

The MCC consists of epidermis, a mucosal surface skeletal muscle (that is superficial to platysma), and glandular tissue. There have been many theories that attempt to explain these findings. Some propose that pressure from the pericardial roof on the developing branchial arches results in pressure necrosis and scarring. Others postulate that rupture of a pathological adhesion between the epithelium of the cardiohepatic fold with that of the ventral part of the first branchial arch causes tissue ischemia, again with localized necrosis and scarring. These theories, however, do not explain the consistent anatomy of the MCC or the presence of skeletal muscle and glandular tissue [8].

Another hypothesis that does help to explain the histological findings is the abnormal development of the first branchial arch. On day 22 in the human embryo, the branchial arches are forming. The first arch is divided into maxillary and mandibular processes with a horizontal cleft dividing each side at the midline. The mandibular process merges on day 26. A delay in this could result in ectodermal cells with underlying mesodermal cells being deposited in the ventral aspect of the neck. These would continue to differentiate and form skeletal muscle (tongue derivative), salivary glandular tissue, and a mucosal surface, thus resulting in the MCC defect [3, 8]. This second theory explains the absence of hair follicles or sweat or sebaceous glands. It is concluded that the skin protuberance could be a vertical outgrowth of tongue muscle, since the fibrous cord is related to the fibrous median septum of the tongue base; salivary glands in the sinus tract and mucoid discharge contribute to the theory. Usually, MCC is associated with a spectrum of midline anomalies related to the branchial arches, including a median cleft of the lower lip and mandible, hypoplasia, or absence of other midline neck structures [2, 4].

MCC is diagnosed by clinical examination, and the main characteristic is a protruding lesion at the midline of the anterior region of the neck, between the chin and the suprasternal notch. This lesion extends caudally as a longitudinal cleft lined by a reddened epithelium that is most commonly desquamative. Usually, a mucus-secreting opening of the fistulous tract can be found in the most caudal portion of the cleft. Palpation reveals a subcutaneous fibrous cord that extends to the entire cleft or part of it, from the submentonian region to the suprasternal notch [3]. The sinus should be probed and the blind end visualized by radiography. In addition, associated abnormalities of the thyroid gland and other related anomalies should be excluded by ultrasonographic examination. The regional site of the sinus tract is important in distinguishing the MCC from a thyroglossal fistula, since the tract extends caudally in the former and cranially in the latter [4].

On histological examination, the lesion consists of keratinized stratified squamous epithelium and fibrous tissue and is characterized by the absence of epithelial adnexa in the dermis. Salivary gland tissue and pseudostratified ciliated columnar epithelium are observed in the fistulous tract and in the cyst. In the area of the nipple-like protuberance, there is normal skin, but cartilage and muscle can also be found [3, 10].

Treatment of MCC involves surgical resection and proper reconstruction. Delay in treatment of MCC changes the growth of the lower third of the face and particularly affects mandibular development and extension of the neck [11]. Reconstruction of the defect needs surgical maneuvers other than primary closure, as the primary closure would lead to an unsightly scar and a vertical scar band that would cause restriction of neck movements. Z-plasty is one of the surgical procedures used to close the defect [12]. Single, double, or multiple Z-plasty is the treatment of choice for MCC [3].

Given the rarity of MCC, we decided to report this case. Our findings are consistent with the literature. Our patient has recovered uneventfully from the surgical treatment.

## Conclusions

MCC is an unusual congenital defect that may be encountered by the otolaryngologist, pediatrician, pediatric surgeon, or other health care professional. Surgical excision and reconstruction of the entire defect with multiple Z-plasty reconstruction yields good cosmetic results while improving neck mobility and reducing the risk for long-term cervical tethering and mandibular defects.
